# N6-methyladenosine modulation classes and immune microenvironment regulation in ischemic stroke

**DOI:** 10.3389/fnmol.2022.1013076

**Published:** 2022-12-23

**Authors:** Hongmiao Tao, Lihua Dong, Lin Li

**Affiliations:** ^1^Medical College, Jinhua Polytechnic, Jinhua, China; ^2^College of Basic Medical Sciences, Zhejiang Chinese Medical University, Hangzhou, China

**Keywords:** ischemic stroke, m6A, immune, class, GEO

## Abstract

N6-methyladenosine (m6A) modifications play an important role in the differentiation and regulation of immune cells. However, research on m6A in ischemic stroke (IS) is still in its infancy, and their role of the immune microenvironment remains unknown. In this study, we systematically assessed the modification classes of m6A regulators in IS based on the GEO database (GSE16561 and GSE22255). We found that in IS patients, IGF2BP2, IGF2BP1, and YTHDF2 expression was significantly upregulated, and ELAVL1, LRPPRC, METTL3, ALKBH5, CBLL1, and METTL14 expression was significantly downregulated. Seven IS-related genes (ELAVL1, IGF2BP2, LRPPRC, YTHDF2, ALKBH5, METTL14, and YTHDC1) were finally screened by logistic and least absolute shrinkage and selection operator (LASSO) regressions, and the AUC of the riskScore was 0.942, which was a good classification. For immune infiltration, there were highly significant differences in memory B cells, CD8 T cells, monocytes, activated dendritic cells, and mast cells between IS and normal samples. The IS samples were grouped into three classes by consistent clustering, and 15 m6A genes were differentially expressed in the different classes. Multiple infiltrating immune cells, immune-associated genes, and HLA-associated genes differed significantly across m6A modification classes, indicating the diversity and complexity of m6A modifications in the immune microenvironment of IS. Finally, 487 genes associated with the m6A modification class were identified, and 227 potential drugs were found. Our findings demonstrated that m6A modification plays a crucial role in the immune regulation of IS.

## Introduction

Stroke is a serious form of cerebrovascular disease, and ischemic stroke (IS) is one of its common subtypes. Studies have shown its prevalence to be as high as 85%, making it one of the leading causes of human mortality (Della-Morte et al., 2012). IS refers to a type of cerebrovascular disease in which the narrowing or occlusion of the blood supplying arteries in the brain leads to ischemic necrosis of cerebral softening of the brain tissue and is the second most common cause of death in the world (Lindsay et al., 2019). Currently, the main clinical treatment for IS thrombolysis or interventional thrombectomy, but both are limited by the narrow time window for treatment and the high risk of bleeding (Schellinger and Köhrmann, 2014). In recent years, many studies had focus on the diagnosis and prognosis of IS. Katharina et al. developed and external validated of a prognostic model for ischemic stroke after surgery (Platzbecker et al., 2021). Another study developed diagnostic model for acute IS based on four circulating microRNAs (miR-125a-5p, miR-125b-5p, and miR-143-3p; Tiedt et al., 2017). Moreover, another pyroptosis-related immune model had been constructed for IS prognosis and its responses to immunotherapy (Shi et al., 2022). The pathophysiological processes following stroke are complex and extensive, and the inflammatory response plays a key role in the pathophysiological processes following ischemic stroke (Fu et al., 2015). Many studies have shown that neuroinflammation following the onset of IS is an important factor in the long-term prognosis of ischemia. The immune system is involved in the whole process of IS, from the mechanisms of action of risk factors to the production of neurotoxicity to tissue repair and remodeling, and the immune system and the brain interact to regulate disease trends. After the onset of IS, a variety of factors, including ROS formation, necrotic cells, and damaged tissue, can cause inflammatory cell activation, resulting in an inflammatory response (Fann et al., 2013; Petrovic-Djergovic et al., 2016). Stroke and immune response are reciprocal to the pathology and time of event and it progresses till untreated (Chavda et al., 2021). The immune reaction during ischemia opens new doors for advanced targeted therapeutics. Nowadays, stem cell therapy has shown better results in stroke-prone individuals (Kawabori et al., 2020). Few monoclonal antibodies like natalizumab have shown great impact on pre-clinical and clinical stroke trial studies (Elkind et al., 2020). Therefore, exploring the effects of the immune system in the development and progression of IS may help reveal the key to the pathological mechanisms behind it and provide a basis for finding potential therapeutic targets.

There are several types of posttranscriptional modifications in RNA, of which N6-methyladenosine (m6A) is one of the most common, accounting for over 50% of all RNA methylation (Sarin and Leidel, 2014). The m6A-related enzymes include a variety of regulatory proteins encoded by writers, erasers, and readers. In mammals, m6A is widely present in a variety of tissues, with high abundance in the kidney, liver, and brain (Chang et al., 2017), and its level peaks in the adult brain (Meyer et al., 2012). Subsequently, studies on the role of m6A in the nervous system began to increase, including those on its role in the regulation of neuronal development and the effects of m6A on neuroplasticity. Chokkalla et al. (2019) showed that m6A levels were significantly higher in the ischemic stroke group than in the sham-operated group, mainly through a reduction in m6A demethylases (e.g., FTO). In ischemia-induced neurological diseases, the homeostasis of the neurovascular unit (NVU) is disrupted, and its treatment and recovery are largely dependent on neurovascular remodeling. Based on studies of the role of RNA methylation in neurological and vascular neogenesis and repair, it is clear that many related molecules have become important targets in the prevention, diagnosis, and treatment of related diseases (Wei et al., 2017). In recent years, studies have also identified an important role for m6A modifications in the differentiation and regulatory functions of immune cells. In 2005, Karikó et al. (2005) found that dendritic cells exposed to RNA modifications such as m6A expressed significantly lower levels of cytokines and activation markers than cells without m6A. In 2017, Li et al. (2017) first reported that m6A affects T-cell homeostasis by targeting signaling molecules in the initial T-cell IL-7/STAT5/SOCS signaling pathway.

A recent study revealed that database mining based on the TCGA and GEO databases has been a promising strategy to identify biomarkers for the diagnosis and therapy of many diseases, including IS and cancer (Li et al., 2020; Fang et al., 2022; Liang et al., 2022; Xu et al., 2022). It is thus clear that m6A modifications play a very important role in immune regulation, yet there is still a lack of systematic exploration of their pathogenesis in IS. In this study, we systematically assessed the modification classes of m6A regulators in IS based on public databases.

## Materials and methods

### Public databases to download ischemic stroke expression data and clinical information

GSE16561 and GSE22255 data were downloaded from the GEO database.^1^ GSE16561 contains 39 IS peripheral blood samples (denoted as IS in this paper) and 24 normal samples (denoted as NC), and GSE22255 contains 20 IS samples and 20 NC samples. Expression data from the two datasets were combined, and batch effects were removed using the R package sva for subsequent analysis.

### m6A regulator engraving

The m6A gene was downloaded from previous literature (Zhang et al., 2021); see Supplementary Table 1 for details. A Circos map of the m6A gene was made using the R package RCircos. A Protein–protein interaction (PPI) network map of m6A was made using STRING.^2^

### Expression levels of m6A regulators

Differential analysis (R package limma) was performed on the expression data of IS and NC samples, following BH calibration. The differential genes were screened according to BH correction *p* value <0.05. Information on m6A genes was extracted to draw volcano plots, boxplots, and heatmaps (R packages ggpubr and pheatmap). The Wilcoxon nonparametric test was performed between groups in boxplots.

### Correlation of m6A regulator expression

Expression data of m6A genes were extracted for IS samples and all samples, correlations between m6A genes were calculated, and correlation plots were drawn. The two genes with the highest correlation were selected to draw scatter plots for the presentation of results.

### Identification of disease-related m6A regulators based on univariate logistic regression and screening of redundant factors by least absolute shrinkage and selection operator (LASSO) regression

A one-way logistic regression model (glm function in the R base package stats) was constructed using m6A gene expression data from IS and NC samples, and genes significantly associated with IS were screened by value of *p* < 0.05. Then, least absolute shrinkage and selection operator (LASSO) regression analysis was carried out using the R package glmnet, with the parameter family = “binomial,” to plot the independent variable coefficients of the trajectories. If all the data are fitted at one time, it may cause overfitting, so the cv.glmnet function was used to perform cross-validation and draw the cross-validation result graph.

### Construction of classifiers based on multifactor logistic regression using the m6A factors after LASSO to assess the diagnostic efficacy

Using the related genes screened in Step 5, a multifactorial logistic regression model was developed as a classifier (riskScore), and receiver operating characteristic (ROC) curves were plotted using the R package pROC.

### m6A regulators and the immune microenvironment

For the IS and NC samples, the percentage of immune cells in the 22 classes was calculated using the R package CIBERSORT, and plotted boxplots were prepared to show the differences in immune cells between the IS and NC groups. The Wilcoxon nonparametric test was used for different statistics. Immune cells were then calculated for correlation with the m6A gene. Similarly, box plots of immune-associated genes (Mak et al., 2016) between the IS and NC groups were plotted, and correlations between immune-associated genes and m6A genes were calculated. The HLA expression data were extracted from the expression data. Boxplots of HLA between the IS and NC groups were plotted, and correlations between HLA and m6A genes were calculated.

### Identification and characterization of m6A regulator-mediated RNA methylation modification classes

Consensus clustering was performed using the R package ConsensusClusterPlus with maxK = 6, reps = 100, pItem = 0.8, pFeature = 1, clusterAlg = “pam,” and distance = “spearman” for the m6A expression data of the IS samples. The IS samples were divided into different subtypes, and boxplots and heatmaps were drawn to show the differences in m6A gene expression among the different subtypes.

### Clinical features of different modification classes

Waffle plots were drawn using each clinical characteristic of the IS sample and the risk score and the Kruskal–Wallis rank-sum test.

### Different modification classes of the immune/inflammatory microenvironment

For the expression data of IS samples, the R package CIBERSORT was used to calculate the proportion of 22 types of immune cells, a boxplot was drawn to show the differences in immune cells between different subtypes, and the Kruskal–Wallis nonparametric test was used to determine significant differences. Boxplots were also drawn to show the differences in immune-related and HLA-related genes between the different subtypes.

### Functional analysis of different modification classes

The Kyoto Encyclopedia of Genes and Genomes (KEGG) dataset (c2.cp.kegg.v7.4.symbols.gmt) was downloaded from the Gene Set Enrichment Analysis (GSEA) database.^3^ For the IS samples, gene set variation analysis (GSVA) was performed using the R package GSVA. The enrichment results for each subtype were then analyzed differentially using the limma package, and the differential enrichment function was selected for heatmap drawing.

### Differentially expressed genes with different modification classes and functional analysis

Differential expression analysis was performed on two combinations of subtypes 1, 2, and 3, and differentially expressed genes (DEGs) were screened for value of *p* < 0.05. The intersection was then taken, and Gene Ontology (GO) and KEGG functional enrichment analyses were performed with the “clusterProfiler” package using the intersecting differential genes. A chart was drawn to show the top 20 enrichment results.

### Identify potential drugs based on network proximity

Through the drugbank drug-target relational database (Peng et al., 2020); drug information was extracted according to the intersection of differential genes obtained in the 12th step. The human protein interaction file was downloaded from STRING,^4^ the information of the intersecting differential genes was extracted, the results were imported into Cytoscape (v3.7.2; Shannon et al., 2003), and the cytoHubba (Chin et al., 2014) plugin was used to calculate the degree of the intersecting differential genes. The minimum required interaction score was set as 0.5.

### Clinical serum specimens and qRT–PCR

After receiving approval from the Ethics Committee of Maoming Petrochemical Hospital and obtaining written informed consent, we collected serum from ischemic stroke patients (*n* = 40) and healthy controls (*n* = 40). Our study was performed following the guidelines outlined in the Declaration of Helsinki. A TRIzol LS isolation kit (Thermo Fisher Scientific, Waltham, MA, United States) was used for the isolation of total mRNAs from serum. Next, cDNA was synthesized by reverse transcription according to the manufacturer’s instructions of the miScript II RT kit (Qiagen, Germany). A sequence of steps was performed with the help of a LightCycler 480 Real-Time PCR System (Roche Diagnostics, Mannheim, Germany) and SYBR Green qPCR kit (SYBR Premix Ex Taq II, TaKaRa) in 96-well plate. The running program of RT–qPCR was set as follows: 95°C for 1 min, then 40 cycles of 95°C for 10 s, 55°C for 30 s, and 70°C for 30 s. GAPDH was used as an internal reference. The fold-changes in gene expression were calculated with the 2^−ΔΔCt^ method.

### Statistical analysis

Chi square test was conducted to analyze clinical data and categorical variables presented as percentages. The distribution of data was checked with the A Shapiro–Wilk test. R-packet limma was used for the difference of gene expression. Wilcoxon rank sum test was used to compare the differences between the two groups, and Kruskal-Wallis rank sum test was used to compare the differences among three groups. The diagnostic model was constructed with stepwise logistic regression analysis. The diagnostic value of gene expression in predicting IS was evaluated with receiver operating characteristic (ROC) curves.

## Results

### m6A regulator engraving

The sample information of GEO datasets GSE16561 and GSE22255 is shown in Table 1. The location of the 23 m6A genes on the chromosome is shown in Supplementary Figure 1A, and the PPI network is shown in Supplementary Figure 1B. There are two erasers, eight writers, and 13 readers.

**Table 1:** Sample information.

Datasets	Accession	Platform	No. of probes	IS samples	NC samples
Microarray	GSE16561	GPL6883	24,526	39	24
	GSE22255	GPL570	54,675	20	20

### Expression levels and correlation of m6A regulators

**Figure 1:**
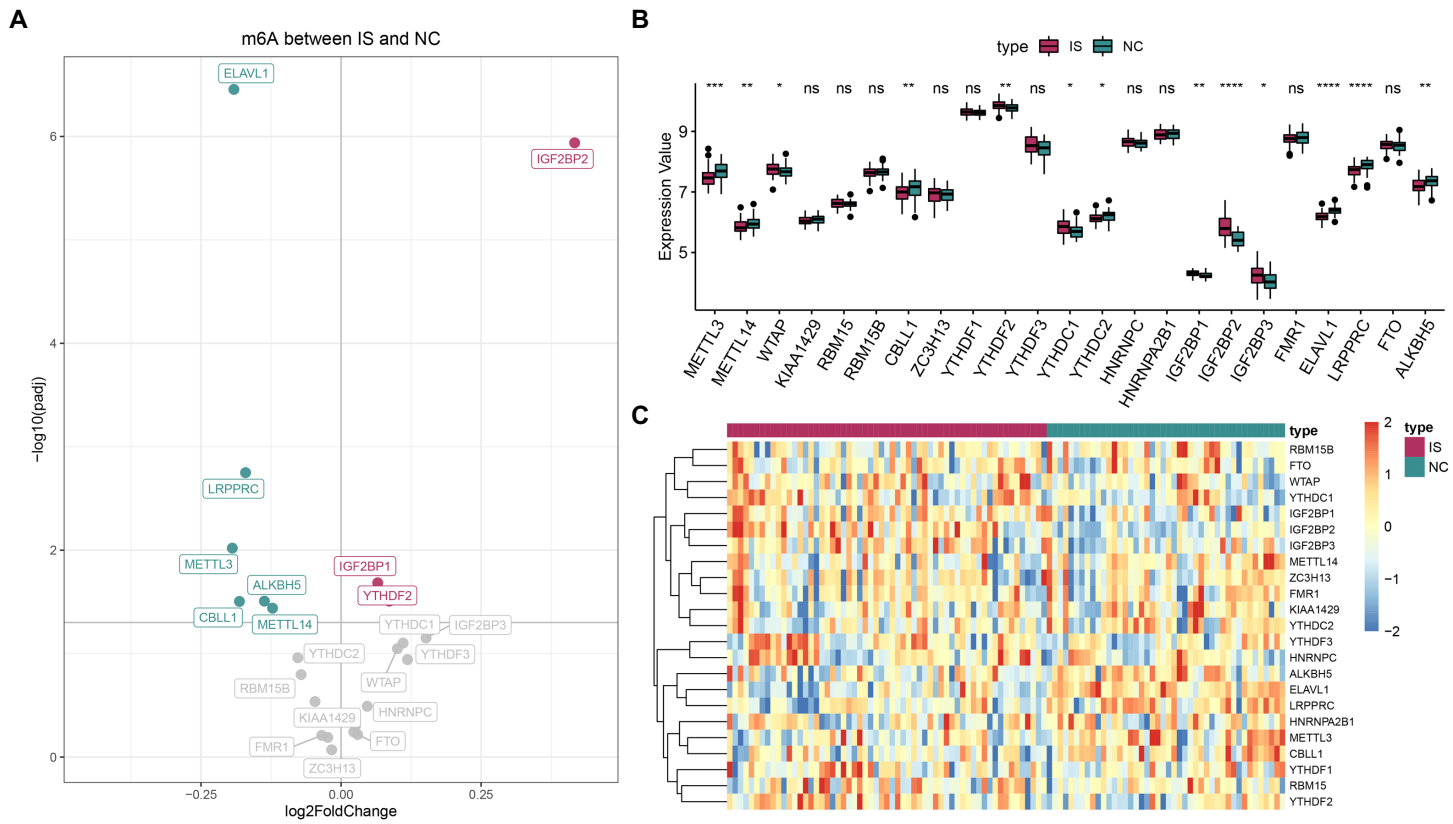
Expression landscape of N6-methyladenosine (m6A) regulators in ischemic stroke. **(A)** Volcano plot of m6A differential genes. **(B)** The boxplot of m6A regulators by the Wilcoxon test. **(C)** Expression heatmap of m6A regulators. ns: value of *p* ≥ 0.05; *: value of *p* < 0.05; **: value of *p* < 0.01; ***: value of *p* < 0.001; and ****: value of *p* < 0.0001.

For IS and NC differential analysis, IGF2BP2, IGF2BP1, and YTHDF2 were upregulated in IS samples, and ELAVL1, LRPPRC, METTL3, ALKBH5, CBLL1, and METTL14 were downregulated in IS samples (Figure 1A). The Wilcoxon nonparametric test showed that 13 m6A genes were significantly differentially expressed between the IS and NC groups (Figure 1B). The heatmap of m6A expression in IS and NC is shown in Figure 1C. The data for plotting the expression heatmap of the m6A gene are detailed in Supplementary Table 2. In the m6A gene expression correlation graph for IS samples or all samples (Supplementary Figures 2A,B), FMR1 and ZC3H13 had the highest correlation, both at 0.78.

### Identifying IS-related m6A regulators, constructing classifiers, and evaluating diagnostic effectiveness

**Figure 2:**
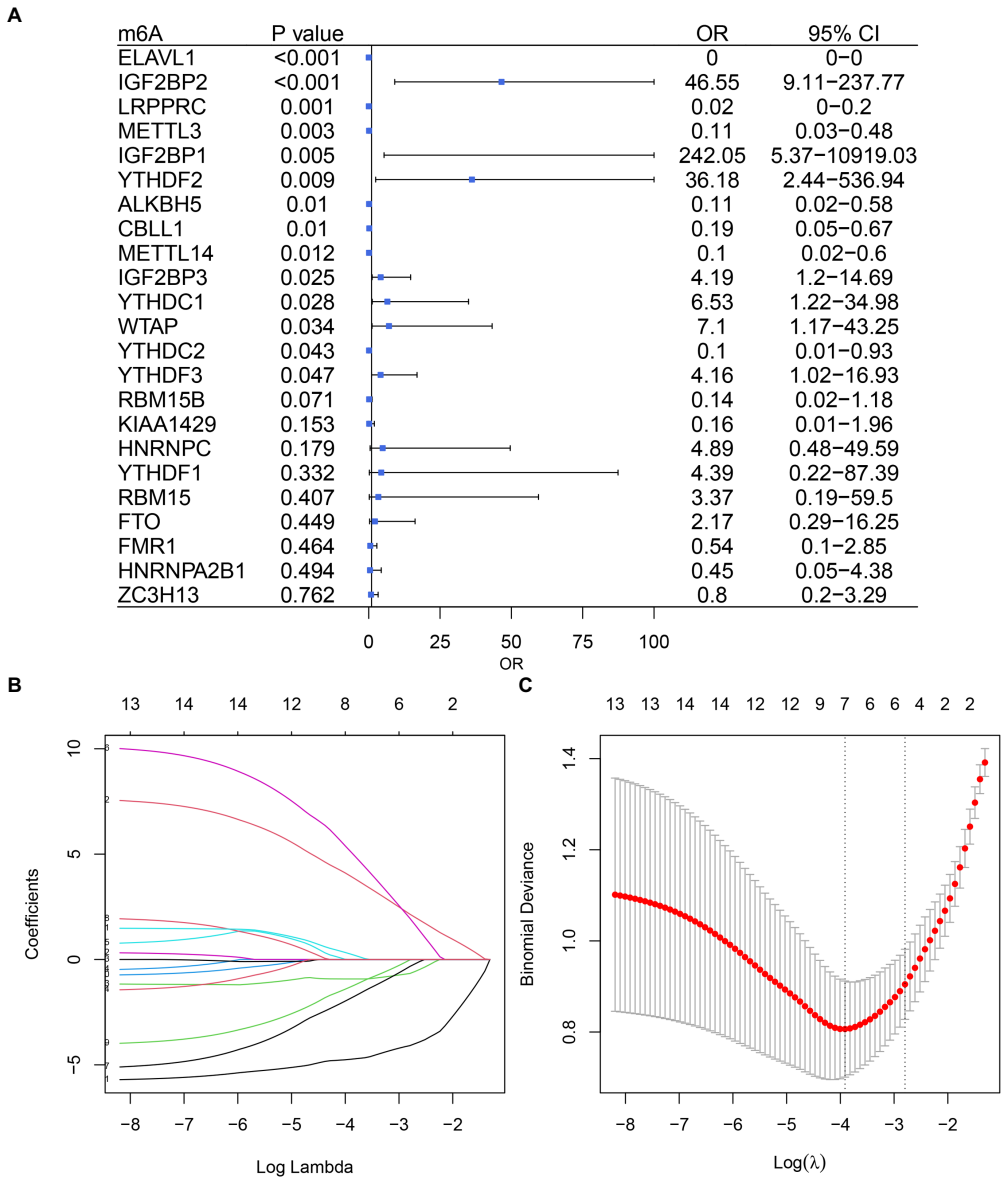
Logistic model based on ischemic stroke and normal sample expression data. **(A)** Univariate Cox regression analysis screened out seven genes associated with IS. **(B)** Profiles of the distribution of LASSO regression coefficients. **(C)** 10-fold cross-validation was used to select the optimal λ value.

**Figure 3:**
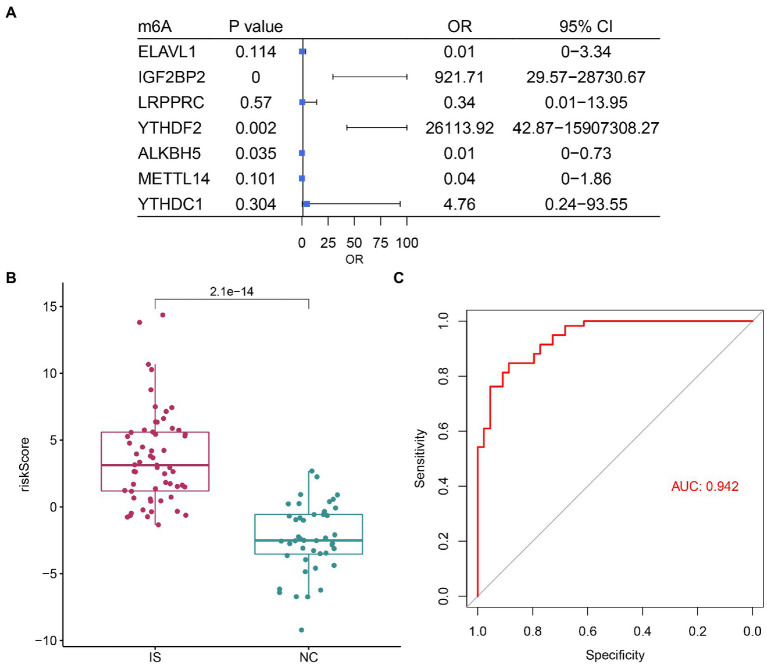
m6A regulators can distinguish ischemic stroke and normal samples. **(A)** Forest map using a multifactorial logistic model with seven IS-related genes. **(B)** The risk distribution between ischemic stroke and normal samples, where ischemic stroke patients have a much higher risk score than healthy patients. **(C)** The AUC value of ROC curve of ischemic stroke and healthy samples by m6A regulators.

Based on univariate logistic regression, seven genes significantly associated with IS were finally screened: ELAVL1, IGF2BP2, LRPPRC, YTHDF2, ALKBH5, METTL14, and YTHDC1 (Figures 2A–C). The results of logistic and LASSO regression models are shown in Supplementary Table 3. Using the m6A regulators after LASSO to construct the classifier (Figure 3A), the risk score of the IS sample was significantly higher than that of the NC sample (Figure 3B), and the AUC of the classifier was 0.942 (Figure 3C). Calculation formula: risk score = −5.034*ELAVL1 + 6.826*IGF2BP2–1.074*LRPPRC + 10.170*YTHDF2−4.293*ALKBH5−3.182*METTL14 + 1.561*YTHDC1. Information on the classifier is detailed in Supplementary Table 4.

### Validation of the expression of m6A-related diagnostic model genes

As shown in Supplementary Figure 3, the expression of ELAVL1, ALKBH5, LRPPRC, and METTL14 (Supplementary Figures 3A–D) was downregulated, while the expression of YTHDF2, YTHDC1, and IGF2BP2 was upregulated in IS compared with healthy controls (Supplementary Figures 3E–G). These results were consistent with those of the GEO dataset.

### m6A regulators and the immune microenvironment

**Figure 4:**
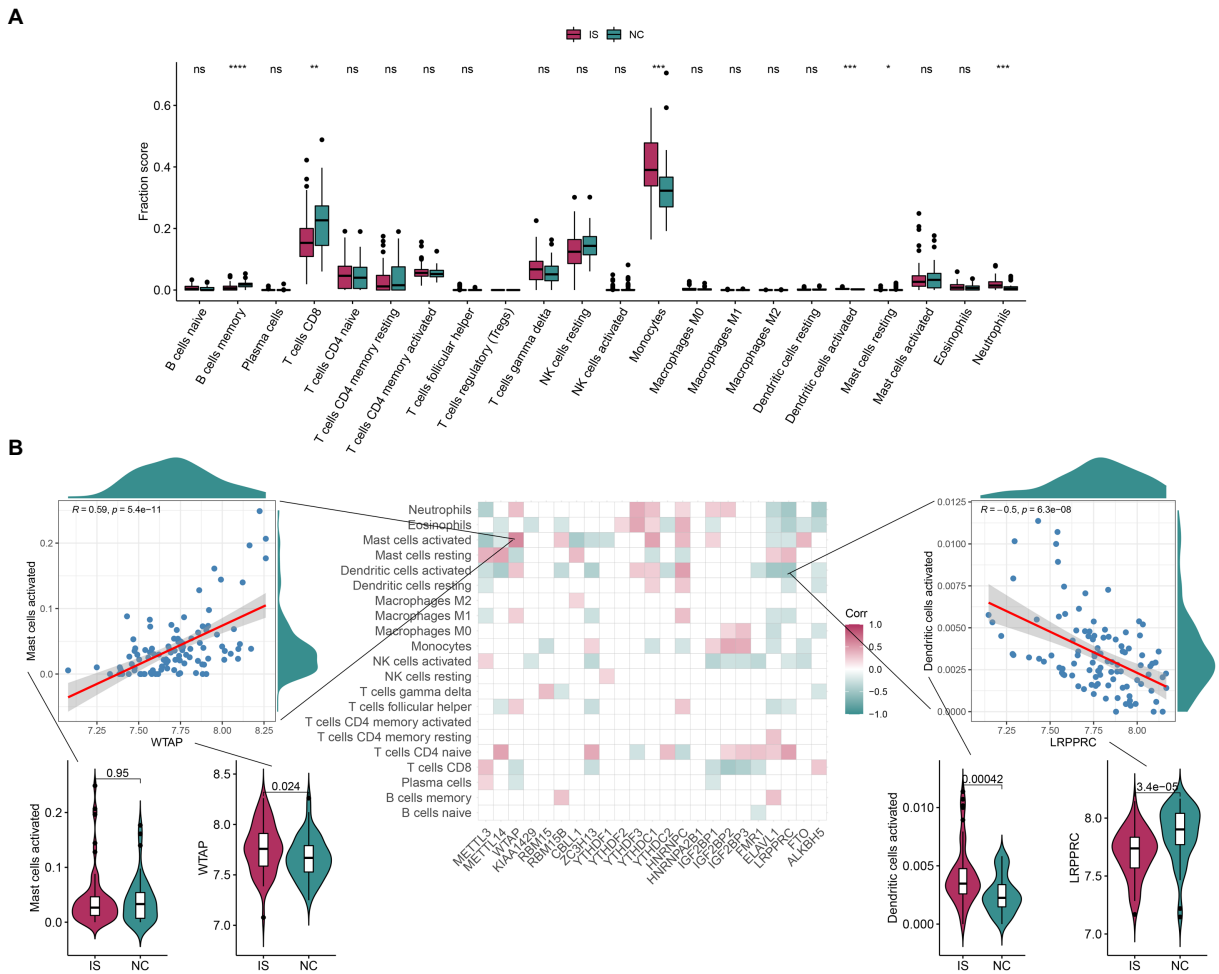
The correlation between infiltrating immune cells and m6A regulators. **(A)** The boxplot of immune infiltrating cell content contains 22 immune cells, each of which is shown in green in the NC group and red in the IS group. Compared with the NC group, the content of CD8 in T cells was significantly lower and the content of Monocytes was significantly higher in IS. **(B)** Correlation plots show the correlation between the degree of immune microenvironment infiltration and each of the m6A regulators. The positive correlation between Mast cells activated and WTAP was high, at 0.59; the negative correlation between Dendritic cells activated and LRPPRC was high, at-0.5. IS, ischemic stroke. NC, normal. ns: value of *p* ≥ 0.05; *: value of *p* < 0.05; **: value of *p* < 0.01; ***: value of *p* < 0.001; ****: value of *p* < 0.0001.

As shown in Figures 4A,B, the differences in the expression of memory B-cells, CD8 T cells, monocytes, activated dendritic cells, resting mast cells, and neutrophils were highly significant between the IS and NC groups, with that of memory B-cells and CD8 T cells being lower in IS and that of monocytes and neutrophils being higher in IS. The positive correlation between activated mast cells and WTAP was high at 0.59, while the negative correlation between activated dendritic cells and LRPPRC was high at-0.5. See Supplementary Table 5 for details of the immune infiltrated cell content. As shown in Supplementary Figures 4A,B, the immune-related genes CD276, ICOS, PDCD1LG2, TNFRSF4, and TNFRSF9 were significantly different between the IS and NC groups. The positive correlation between TNFSF4 and IGF2BP3 was higher at 0.77, while the negative correlation between IL10 and LRPPRC was higher at-0.67. Ten HLA-related genes were significantly different between the IS and NC groups (Supplementary Figure 5A). The positive correlation between HLA-G and YTHDF3 was higher at 0.64, while the negative correlation between HLA-G and LRPPRC was higher at −0.66 (Supplementary Figure 5B).

### Identification and characterization of m6A regulator-mediated RNA methylation modification classes

**Figure 5:**
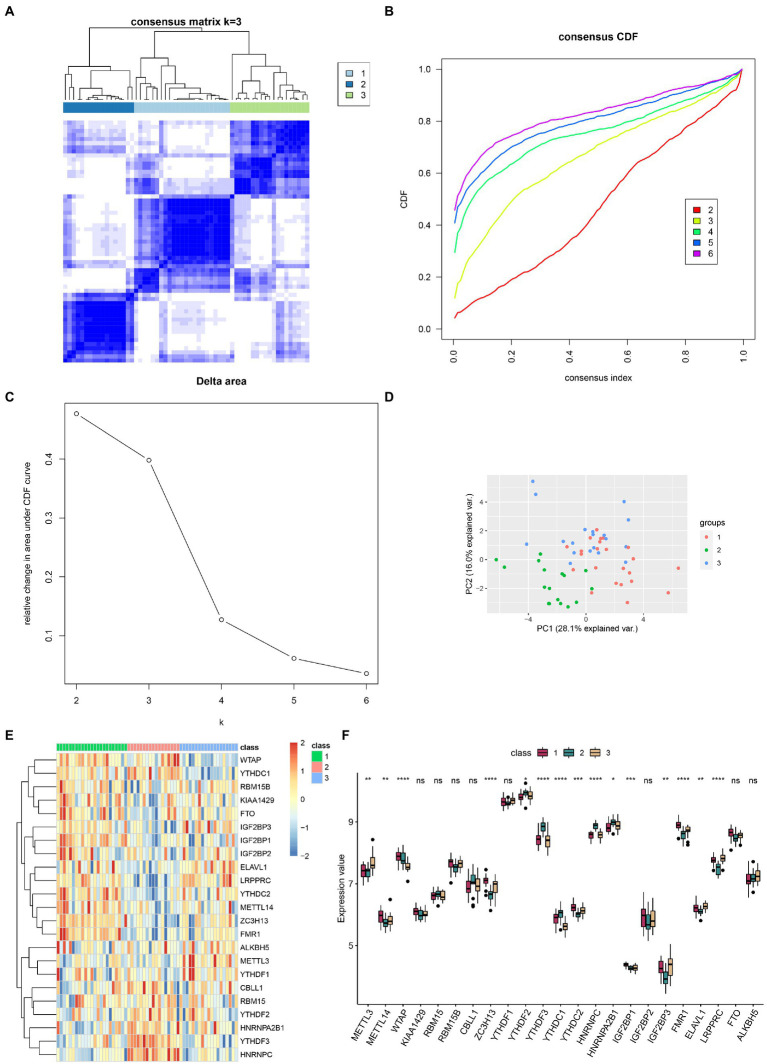
The three different subtypes of m6A regulator-mediated RNA methylation modification classes in IS were identified by unsupervised clustering of 23 m6A regulators. **(A)** Heatmap of the consistency matrix. **(B)** The cumulative distribution curve, with the gentlest slope of the curve decline, FIGURE 5 (Continued)indicates that the best classification was reached. **(C)** The delta area curve. **(D)** The two-dimensional distribution map of the PCA analysis of the IS samples suggested that the three subtypes were well classified. **(E)** Heatmap of m6A regulators expression in different m6A modification classes. **(F)** Boxplot of m6A gene expression in different subtypes, with 15 m6A genes differentially expressed in different m6A modification classes. ns: value of *p* ≥ 0.05; *: value of *p* < 0.05; **: value of *p* < 0.01; ***: value of *p* < 0.001; and ****: value of *p* < 0.0001.

Based on the consensus CDF and delta area, we could separate IS cases into three clusters according to the expression pattern of m6A regulators. The IS samples were divided into three m6A modification classes (numbered 1, 2, and 3); 23 IS samples were classified as category 1, 17 IS samples as category 2, and 19 IS samples as category 3 (Figures 5A–D). For detailed information, see Supplementary Table 6. Fifteen m6A genes were differentially expressed in different m6A modification classes (Figures 5E,F). There is no significant difference between age, gender, and classification and the risk score of the model (Supplementary Figures 6A–D).

### Different m6A modification classes of the immune/inflammatory microenvironment

**Figure 6:**
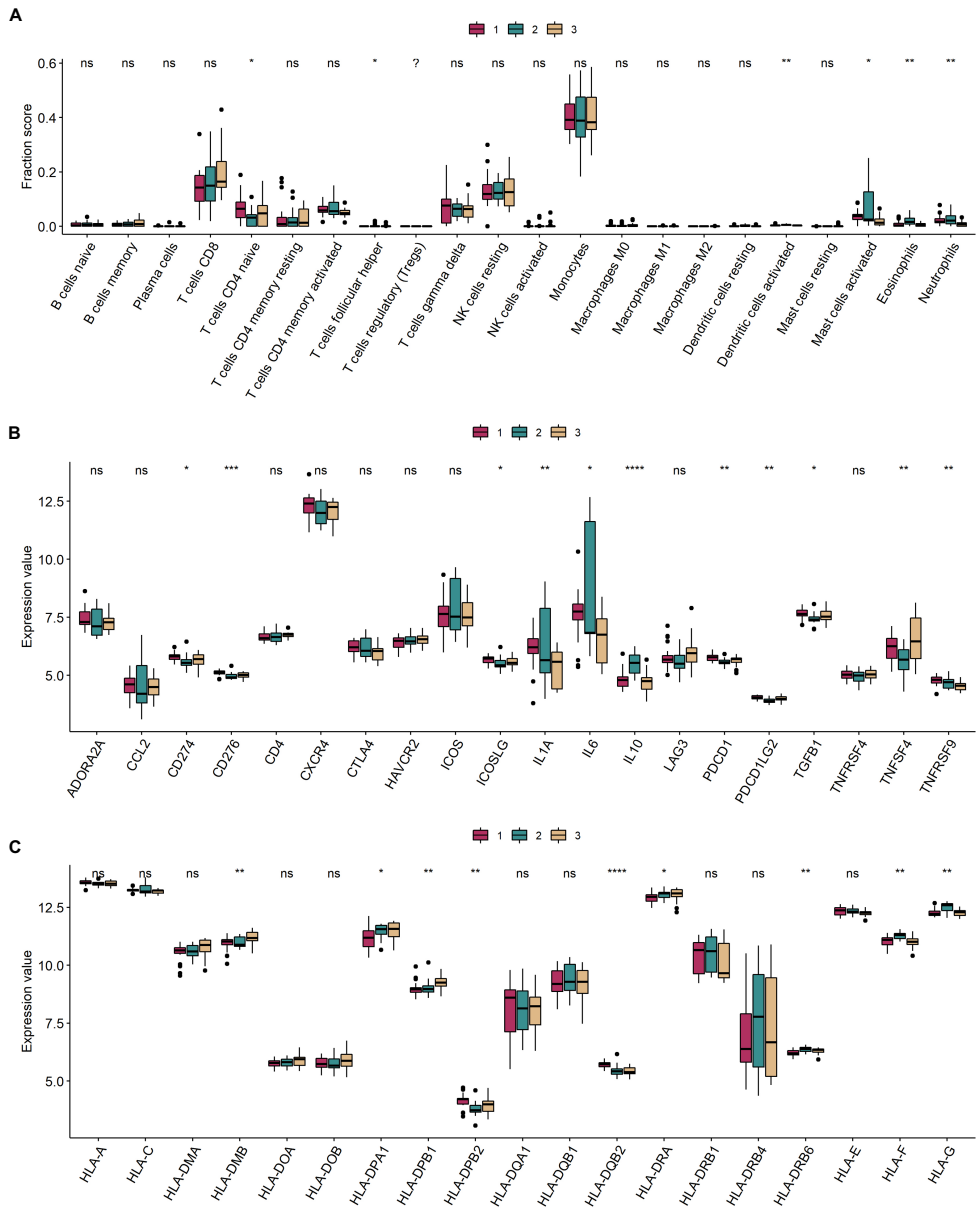
Immune microenvironment between different m6A modification classes. **(A–C)** Boxplots of immune infiltrating cells, immune-related genes, and HLA-related genes in different m6A modification classes. ns: value of *p* ≥ 0.05; *: value of *p* < 0.05; **: value of *p* < 0.01; ***: value of *p* < 0.001; and ****: value of *p* < 0.0001.

Levels of CD4 naive T cells, follicular helper T cells, activated dendritic cells, activated mast cells, eosinophils, and neutrophils were significantly different in the different m6A modification classes (Figure 6A). Information on immuno-infiltrating cells is detailed in Supplementary Table 7. Of these, 11 immune-related genes were differentially expressed in different modification classes (Figure 6B). Nine HLA-related genes were differentially expressed in different modification classes (Figure 6C).

### Functional analysis of the m6A modification classes

**Figure 7:**
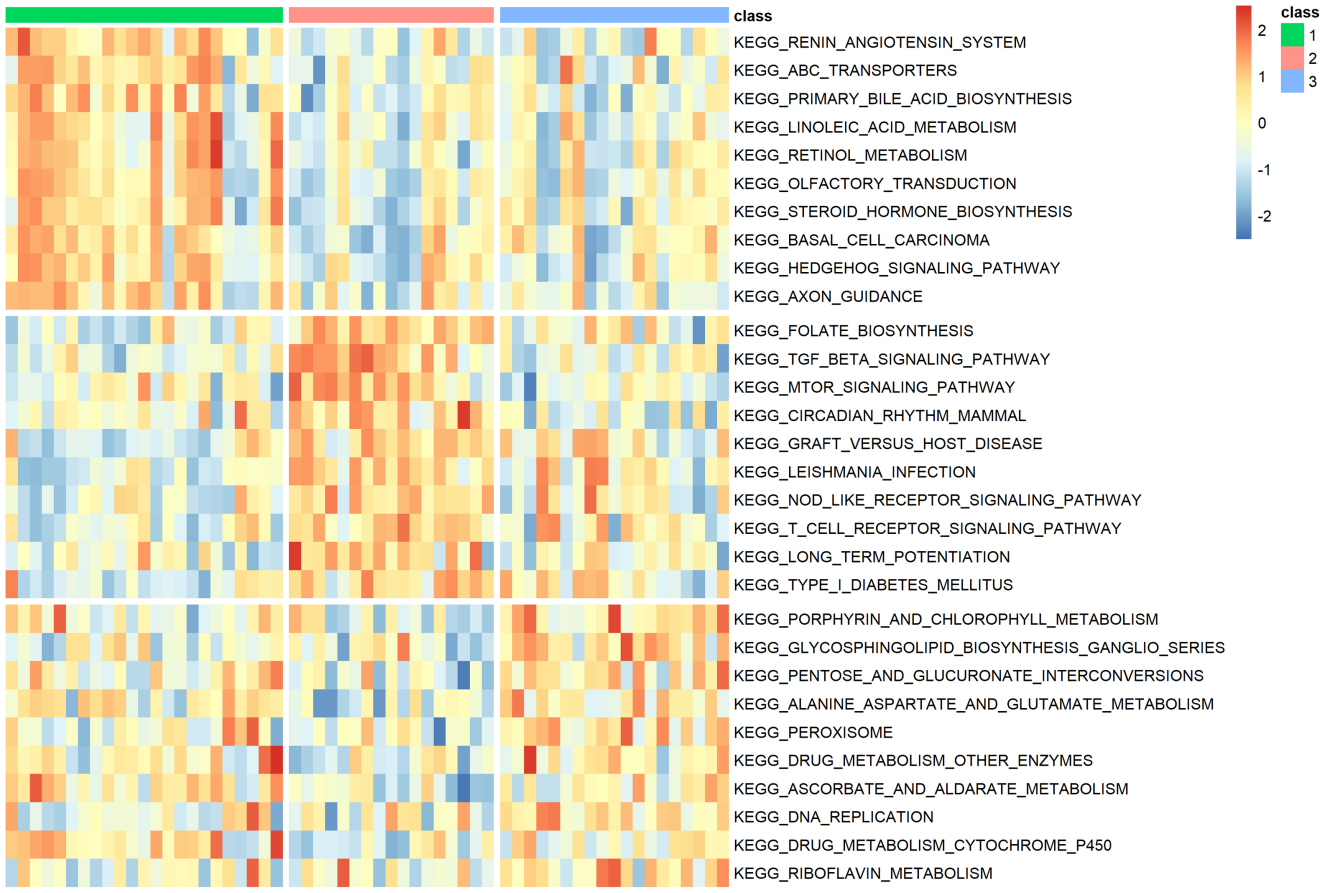
Venn diagram of differentially expressed genes between m6A modification classes.

**Figure 8:**
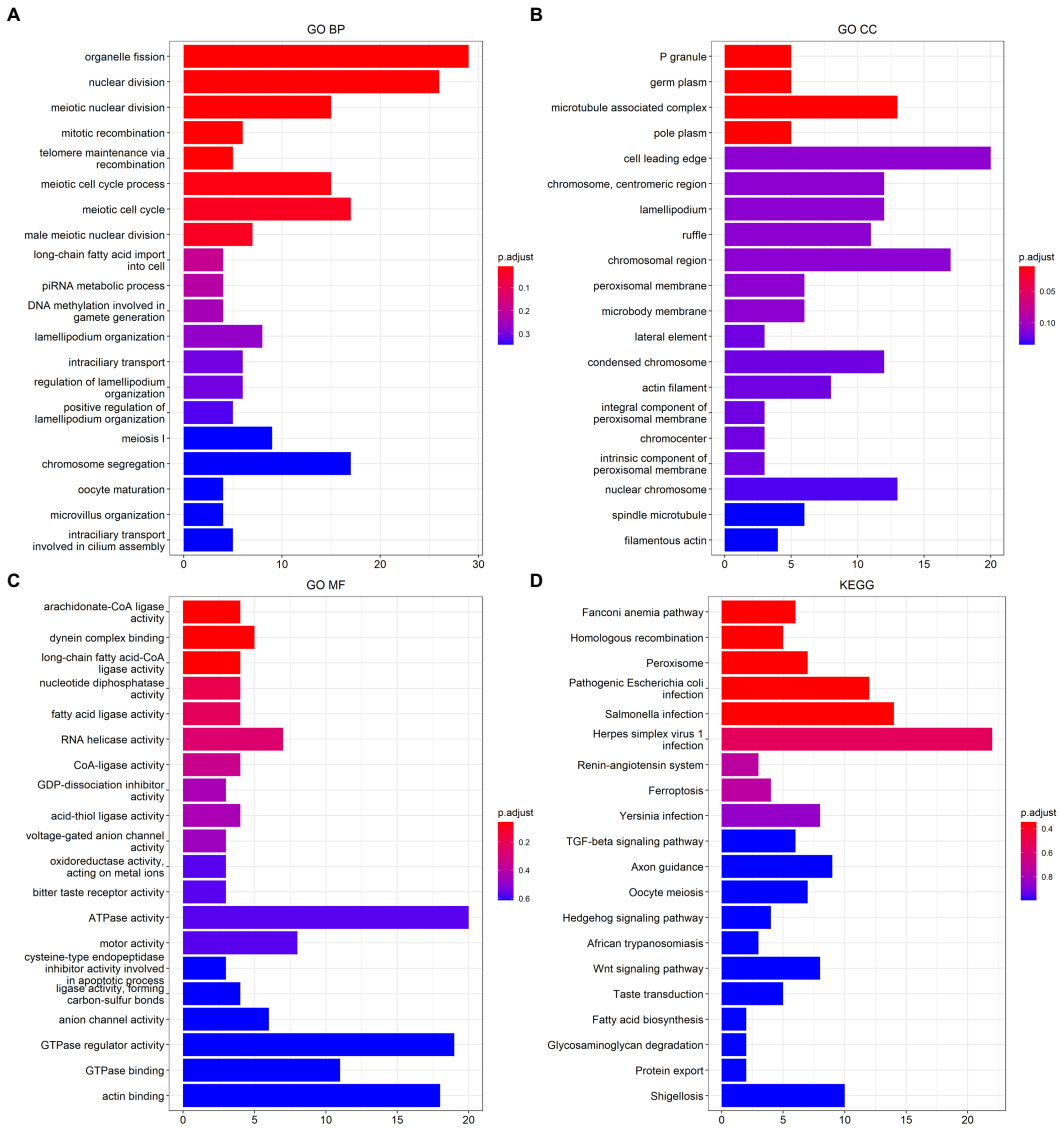
Function analysis of m6A modification classes-related differentially expressed genes in ischemic stroke. **(A)** BP of GO enrichment analysis. **(B)** CC of GO enrichment analysis. **(C)** MF of GO enrichment analysis. **(D)** KEGG pathway enrichment analysis. BP, biological process. CC, cellular component. MF, molecular function. GO, Gene Ontology. KEGG, Kyoto Encyclopedia of Genes and Genomes.

The results of the functional analysis of the different classes are detailed in Figure 7. The first 10 enriched pathways were concentrated in pattern 1, the middle 10 in pattern 2, and the last 10 in pattern 3. For more details, see Supplementary Table 8. A total of 9,004 DEGs were found between classes 1 and 2, 6,819 DEGs were found between subtypes 1 and 3, and 3,201 DEGs were found between classes 2 and 3. A total of 3,201 differentially expressed genes were found between classes 2 and 3. There were 487 overlapping DEGs (Supplementary Figure 7). The first 20 items enriched by GO and KEGG are shown in Figures 8A–D. The enriched GO biological process (BP) was organelle fission and nuclear division, the cellular component (CC) was P granule and germ plasm, and the molecular function (MF) was arachidonate-CoA ligase activity and dynein complex binding. The KEGG pathways were the Fanconianaemia pathway and homologous recombination. Overlapping DEGs and complete enrichment results are shown in Supplementary Table 9.

### Identifying potential drugs based on network proximity

**Figure 9:**
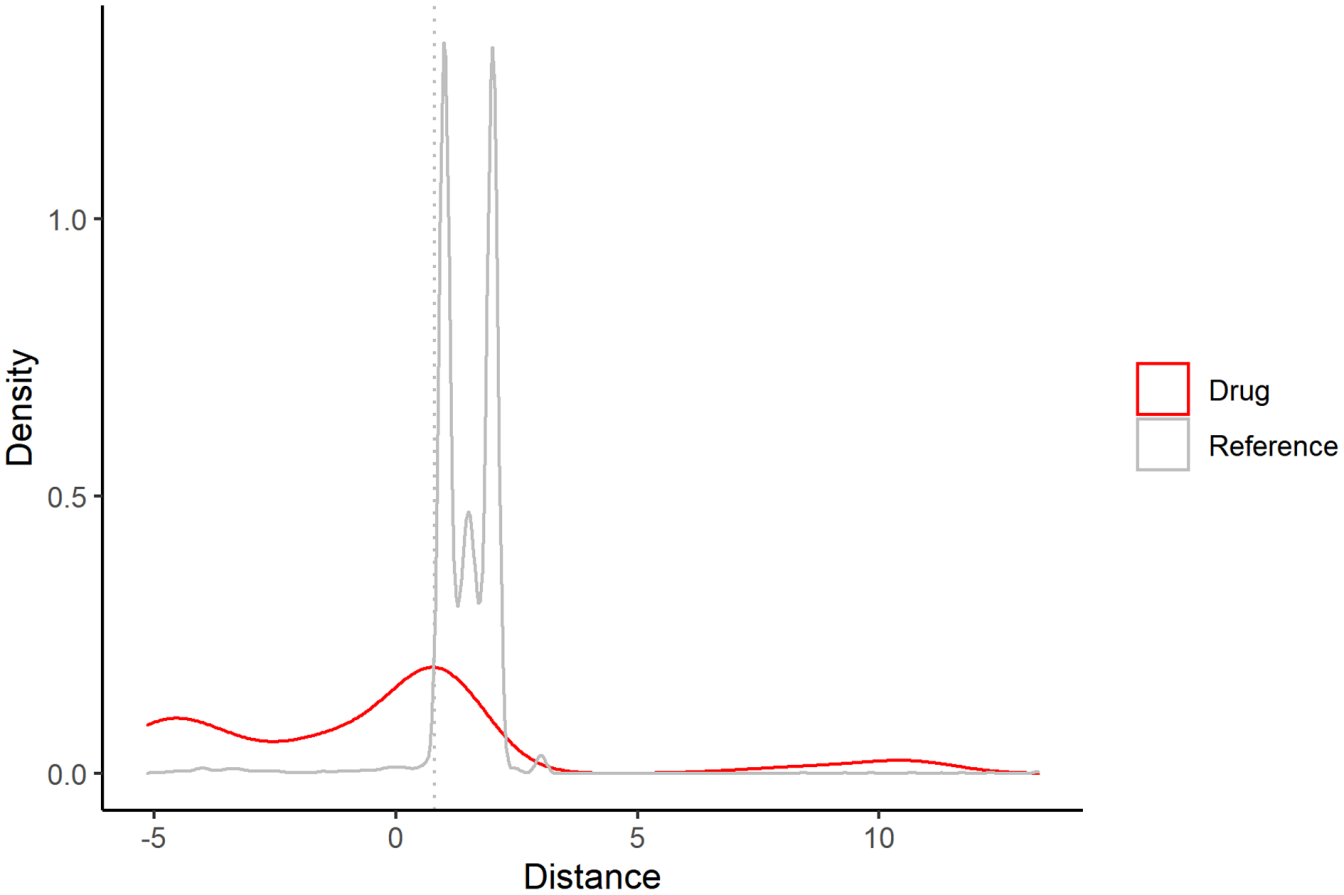
Distance density map of 484 drugs and ischemic stroke.

Figure 9 shows that when the distance is in the interval ≥ 0, the drug and reference overlap, and this part of the drug needs to be excluded. Finally, 227 potential drug information items are obtained; see Supplementary Table 10 for details.

## Discussion

Ischemic stroke (IS) has a very high rate of disability, death, and recurrence. Recent studies have confirmed that the inflammatory response and the intrinsic and peripheral immune responses of the central nervous system (CNS) play a key regulatory role in the overall pathogenesis of IS (Shi et al., 2019). Severe cerebral ischemia following stroke can induce an initial immune-inflammatory response that exacerbates neurological deficits and leads to alterations in the systemic immune system, producing immunosuppression. This ultimately increases the patient’s susceptibility to infection, which in turn leads to an increase in fatal events (Malone et al., 2019). Currently, m6A, the most common and abundant mRNA posttranscriptional modification in eukaryotic cells, has received much attention from researchers. A growing body of evidence confirms the important role of m6A in the differentiation and regulatory functions of immune cells (Han et al., 2019; Li et al., 2022). Thus, our study also observed that m6A modifications play an important role in the regulation of the immune microenvironment in IS.

First, we downloaded the transcriptome expression profiles and corresponding clinical data of IS patients from the GEO database. Through differential expression analysis, it was found that the expression of most m6A regulators was altered between the normal group and ischemic stroke patients; IGF2BP2, IGF2BP1, and YTHDF2 expression was significantly upregulated in IS samples, while ELAVL1, LRPPRC, METTL3, ALKBH5, CBLL1, and METTL14 expression was significantly downregulated in IS samples. Second, based on univariate and multivariate logistic regression analyses, ELAVL1, IGF2BP2, LRPPRC, YTHDF2, ALKBH5, METTL14, and YTHDC1 were finally screened for significant associations with IS. Chokkalla et al. (2019) found increased levels of m6A modifications in the total RNA of middle cerebral artery occlusion (MCAO) mice (subjected to transient middle cerebral artery occlusion). They also found that differentially expressed m6A transcripts play an important role in pathophysiology following IS, such as inflammation, apoptosis, and transcriptional regulation. Similarly, in a study of Yi et al. (2021), m6A regulator expression levels were found to be significantly increased in the MCAO/R rat model compared to the sham-operated group. In addition, in a study of the RNA modification landscape in carotid atherosclerosis (AS), Quiles-Jiménez et al. (2020) found significantly increased total levels of WTAP, METTL3, YTHDF2 and FTO in advanced AS lesions compared to early AS lesions. This suggests that aberrant m6A regulators may be involved in the pathophysiological processes after IS.

In neuroinflammation, m6A modifications have regulatory inflammatory effects, including preventing excessive inflammatory responses or proinflammatory effects (Yu et al., 2019; Wen et al., 2020). Our study also showed that m6A regulators are closely associated with the immune microenvironment of IS, including infiltrating immune cells, immune-related genes, and HLA-related genes. Compared with the normal group, the IS group had a higher content of monocytes and neutrophils. A large number of monocyte-derived macrophages may appear at the site of ischemia 3–7 days after the onset of IS (Breckwoldt et al., 2008). Clinical studies have shown that the CD14+ CD16-and CD14+ CD16+ subpopulations of monocytes increase significantly between 0 and 16 days after the onset of IS, with an increase in the CD14+ CD16-subpopulation being closely associated with tissue damage in the acute and subacute phases of stroke (Kaito et al., 2013). Several studies have shown that Ly6C+ CCR2+ proinflammatory monocytes are predominantly recruited to the ischemic site during the initial phase of IS, but blocking Ly6C+ cell infiltration with CCR2 antagonists or specifically depleting Ly6C+ cells exacerbates IS brain injury and increases hemorrhagic transformation around the infarct (Gliem et al., 2012; Chu et al., 2015). Neutrophils are thought to be the first peripheral immune cells to migrate to damaged brain tissue. Between 0.5 and 6 h after the onset of IS, neutrophil expressing Ly6G and myeloperoxidase begin to migrate and appear in the soft meninges, followed by a gradual infiltration into the perivascular space and superficial layers of the cortex over 1–3 days, eventually appearing widely and peaking in and around the infarct site (Jickling et al., 2015). Studies have shown that neutrophils infiltrating the lesion exert neurotoxic effects in several ways that exacerbate brain damage. For example, activation of neutrophils can release various proinflammatory factors, such as TNF-α, IL-6, IL-1β, monocyte chemoattractant protein-1 (MCP-1), and matrix metalloproteinases (MMPs), which can exacerbate brain injury (Allen et al., 2012). In addition, m6A regulator correlation analysis with immune cell infiltration revealed a significant positive correlation between IGF2BP1 and IGF2BP2 expression and the degree of monocyte and neutrophil infiltration. Xie et al. (2019) found that IGF2BP1 promoted LPS-induced activation of human macrophages and monocytes. Dendritic cells induce activation and differentiation of Naive T cells by presenting antigenic peptides *via* MHC molecules. In our study, we found that the level of Dendritic cells was higher in IS group. The expression of LRPPRC was lower in IS group and negatively correlated with the level of dendritic cells. Thus, LRPPRC may inhibit the immune response in IS process by suppressing Dendritic cells activation. Interestingly, we also found significant differences in the immune-related genes CD276 (PD-1), ICOS, PDCD1LG2 (PD-L2), TNFRSF4, and TNFRSF9 between the IS and NC groups. Among them, CD276 (PD-1), PDCD1LG2 (PD-L2), and TNFRSF9 were more highly expressed in IS. Previous studies have confirmed that coligation of PD-1 with PDL1 or PDL2 triggers inhibitory signals and plays a key role in immune tolerance (Seifert et al., 2019). Increased expression of PD-1 and PD-L was found in the brain tissue of MCAO mice, with the ability to inhibit inflammatory T-cell activation (Ren et al., 2011), reduce the release of cytotoxic proteins from T lymphocytes and avoid neurological damage (Mracsko et al., 2014; Fan et al., 2020). This suggests that immune and m6A-mediated inflammatory responses play a key regulatory role in the IS process, with a variety of immune cells and inflammatory mediators involved in the process of IS injury or neurological repair.

In addition, based on m6A expression data from IS samples, we revealed three distinct classes of m6A methylation modification, and 15 m6A genes were differentially expressed in these classes. Regarding the immune microenvironment, multiple infiltrating immune cells, immune-related genes, and HLA-associated genes differed significantly across m6A modification classes. Compared with other classes, m6A class 1 has more naive CD4 T cells, activated mast cells, and neutrophils. The immune-related genes CD274, CD276, ICOSLG, IL1A, IL6, PDCD1, PDCD1LG2, TGFB1, and TNFRSF9 were significantly expressed, which led to a more active immune state. Thus, m6A methylation modification patterns play a nonnegligible role in immunity, and m6A class 1 patients may be better candidates for immunotherapy. The present study also found that differences in the mRNA transcriptome between different m6A modification classes were significantly associated with the biological pathways ferroptosis, fatty acid biosynthesis, and TGF-beta signaling. Iron death has been shown to play an important role in the pathology of neurodegenerative diseases, ischemia–reperfusion, stroke, and traumatic brain injury (Stockwell et al., 2017). Numerous studies have now revealed that stroke leads to iron overload and disturbances in lipid metabolism, which can trigger iron death and that inhibition of iron death can reduce stroke damage (Tuo et al., 2017; Zille et al., 2017; Alim et al., 2019). In the TGF-beta signaling pathway, TGF-beta1 may upregulate Bcl-2 expression *via* Smad3, thereby inhibiting neuronal apoptosis and increasing ischemic injury (Zhu et al., 2017).

Admittedly, this study also has some drawbacks. First, sample size used to validate this study was small and its practical clinical application is limited. Second, this study is only a preliminary investigation of the role of m6A regulators in IS regulation and cannot provide a complete picture of the molecular mechanisms underlying IS development and evolution.

## Conclusion

This study systematically analyzed the relevance of m6A RNA methylation regulators to immune infiltration in IS based on the GEO database. However, there are some limitations in this study, and the next step will be to conduct basic experiments with a larger sample size to validate the results of this study to further elucidate the role of m6A RNA methylation regulators in the immune microenvironment of IS.

## Data availability statement

The original contributions presented in the study are included in the article/Supplementary material; further inquiries can be directed to the corresponding author.

## Ethics statement

The studies involving human participants were reviewed and approved by Maoming Petrochemical Hospital. The patients/participants provided their written informed consent to participate in this study.

## Author contributions

HT conceived and designed the study and critically reviewed the manuscript. LD performed literature search and analyzed the data and generated the figures and tables. LL wrote the manuscript. All authors contributed to the article and approved the submitted version.

## Funding

This work is supported by Zhejiang Provincial Natural Science Foundation of China (Grant No. LY19H090005); Science and Technology Project of Jinhua City (grant no. 2018-3-025); the Zhejiang Basic Public Welfare Research Program (grant no. LGF20H090006); and the second batch of research project to National Vocational Education teachers‘ innovative team (grant no. ZH2021070301).

## Conflict of interest

The authors declare that the research was conducted in the absence of any commercial or financial relationships that could be construed as a potential conflict of interest.

## Publisher’s note

All claims expressed in this article are solely those of the authors and do not necessarily represent those of their affiliated organizations, or those of the publisher, the editors and the reviewers. Any product that may be evaluated in this article, or claim that may be made by its manufacturer, is not guaranteed or endorsed by the publisher.

## Footnotes

^1^ https://www.ncbi.nlm.nih.gov/gds

^2^ https://cn.string-db.org/

^3^ http://www.gsea-msigdb.org/gsea/index.jsp

^4^ https://cn.string-db.org/

## Supplementary material

Supplementary Figure 1 Landscape of m6A regulators in ischemic stroke. **(A)** The Circos diagram of the 23 m6A genes. **(B)** The PPI network of 23 m6A genes.

Supplementary Figure 2 Correlation of m6A regulator expression. **(A,B)** Correlation diagram of m6A gene between IS samples and all samples, respectively, with darker colors indicating stronger correlations and spaces indicating statistical *p* ≥ 0.05 and not reaching significance levels.

Supplementary Figure 3 The expression of the M6A-related diagnostic model genes. **(A-D)** The expression of ELAVL1, ALKBH5, LRPPRC, METTL14 were downregulated while **(E-G)** the expression of YTHDF2, YTHDC1 and IGF2BP2 was upregulated in IS compared with healthy controls. *: value of *p* < 0.05; **: value of *p* <0.01; ***: value of *p* <0.001.

Supplementary Figure 4 The correlation between immune-related genes and m6A regulators. **(A)** The boxplot of the immune-related genes by the Wilcoxon test. There were significant differences in CD276, ICOS, PDCD1LG2, TNFRSF4, and TNFRSF9 between IS and NC groups. **(B)** Correlation plots show the correlation between the immune-related genes and each of the m6A regulators. The positive correlation between TNFSF4 and IGF2BP3 was higher at 0.77. The negative correlation between IL10 and LRPPRC was high at -0.67. IS, ischemic stroke. NC, normal. ns: value of *p* ≥ 0.05; *: value of *p* < 0.05; **: value of *p* < 0.01; ***: value of *p* < 0.001; ****: value of *p* < 0.0001.

Supplementary Figure 5 The correlation between HLA-related genes and m6A regulators. **(A)** The boxplot of the HLA-related genes by the Wilcoxon test. There were significant differences in HLA−C, HLA−DOA, HLA−DOB, HLA−DPB1, HLA−DQA1, HLA−DQB1, HLA−DRB1, HLA−E, HLA−F, and HLA−G between IS and NC groups. **(B)** Correlation plots show the correlation between the HLA-related genes and each of the m6A regulators. The positive correlation between HLA-G and YTHDF3 was higher at 0.64, while the negative correlation between HLA-G and LRPPRC was higher at −0.66.HLA, human leukocyte antigen. IS, ischemic stroke. NC, normal. ns: p-value ≥ 0.05; *: value of *p* < 0.05; **: value of *p* < 0.01; ***: value of *p* < 0.001; and ****: value of *p* < 0.0001.

Supplementary Figure 6 The clinical characteristics of the situation between the different risk score **(A–C)** and m6A modification classes **(D)**.

Supplementary Figure 7 Potential bifunctional features between the three m6A modification classes.
