# Supplementary figures and images for: N6-methyladenosine modulation classes and immune microenvironment regulation in ischemic stroke
Source: Front Mol Neurosci. 2022 Dec 23;15:1013076. doi: 10.3389/fnmol.2022.1013076 (PMC9907088; doi:10.3389/fnmol.2022.1013076)

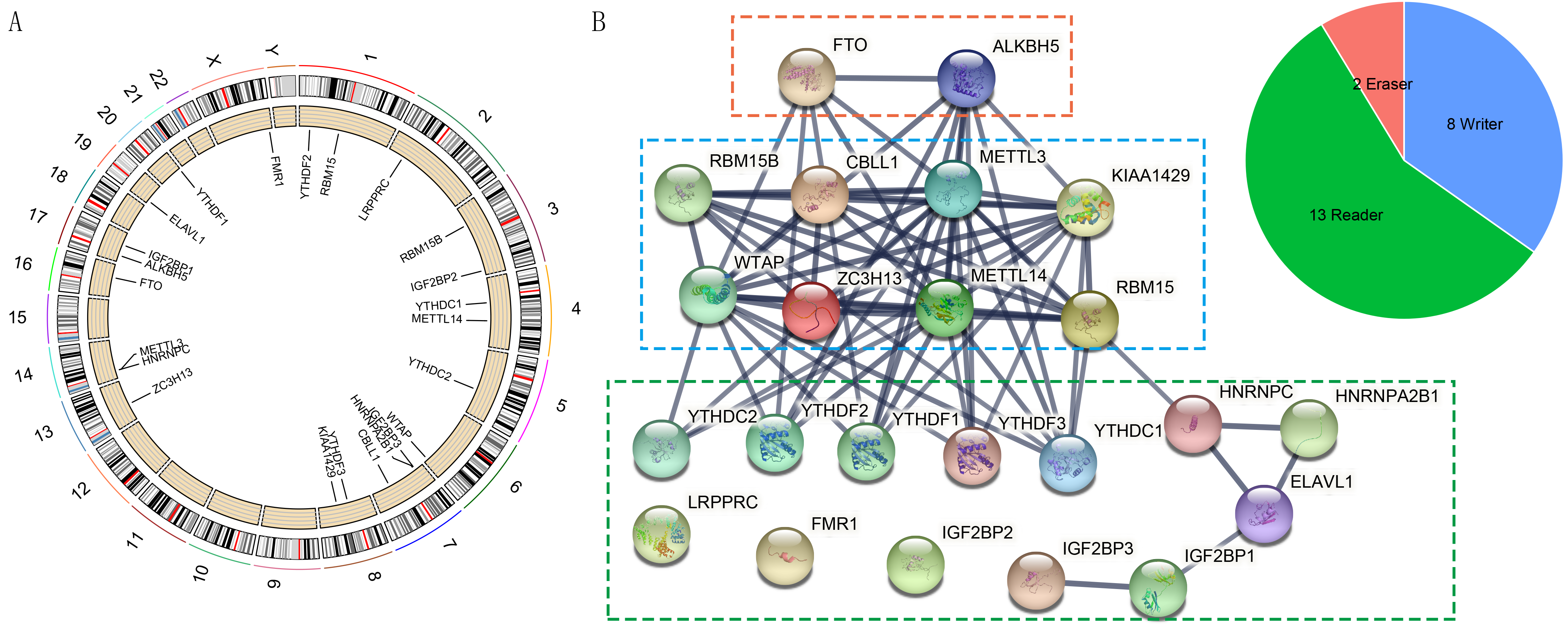

Supplement: Supplementary Figure 1 — Landscape of m6A regulators in ischemic stroke. (A) The Circos diagram of the 23 m6A genes. (B) The PPI network of 23 m6A genes. [file Image_1.TIF]

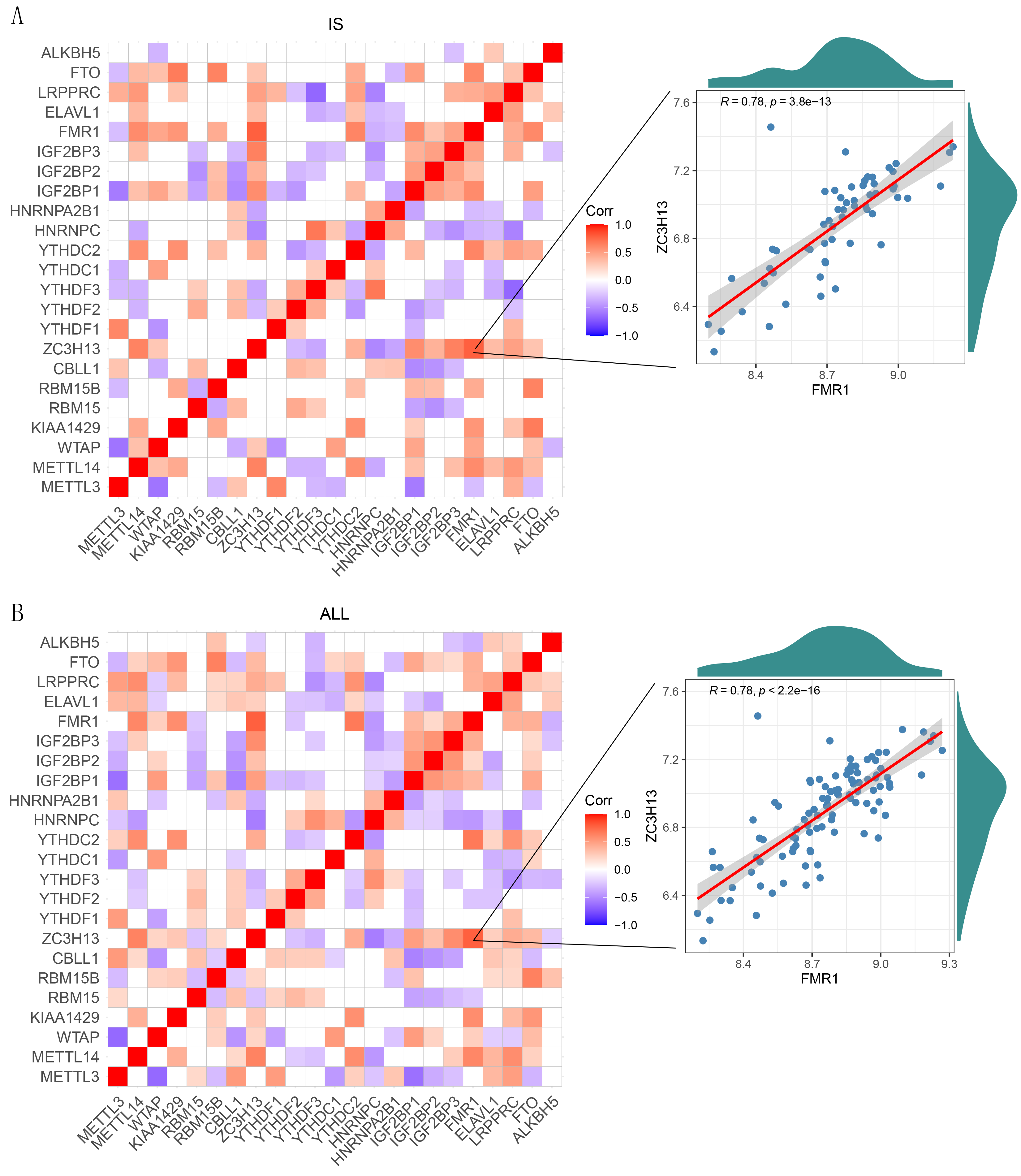

Supplement: Supplementary Figure 2 — Correlation of m6A regulator expression. (A,B) Correlation diagram of m6A gene between IS samples and all samples, respectively, with darker colors indicating stronger correlations and spaces indicating statistical p ≥ 0.05 and not reaching significance levels. [file Image_2.TIF]

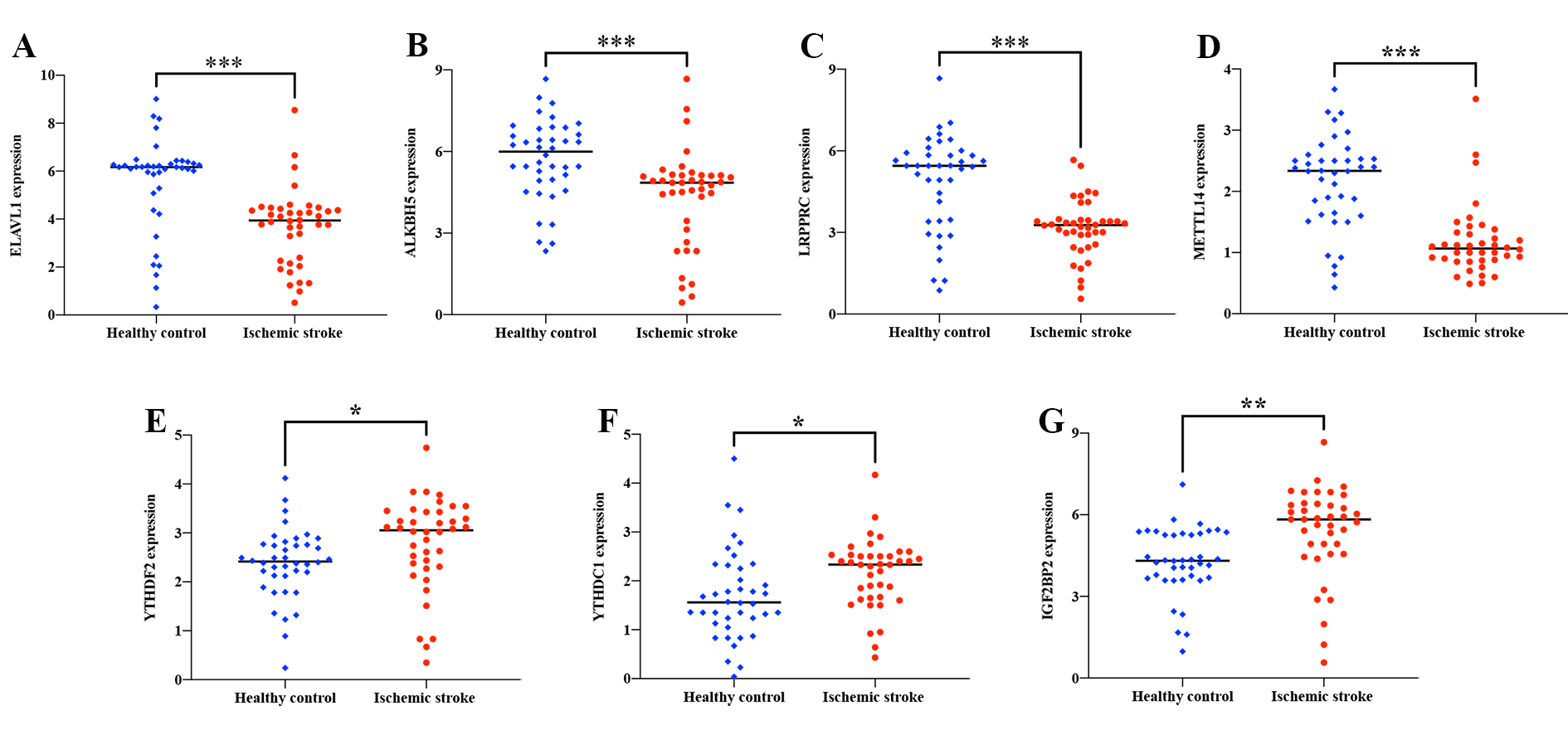

Supplement: Supplementary Figure 3 — The expression of the M6A-related diagnostic model genes. (A-D) The expression of ELAVL1, ALKBH5, LRPPRC, METTL14 were downregulated while (E-G) the expression of YTHDF2, YTHDC1 and IGF2BP2 was upregulated in IS compared with healthy controls. *: value of p < 0.05; **: value of p <0.01; ***: value of p <0.001. [file Image_3.TIF]

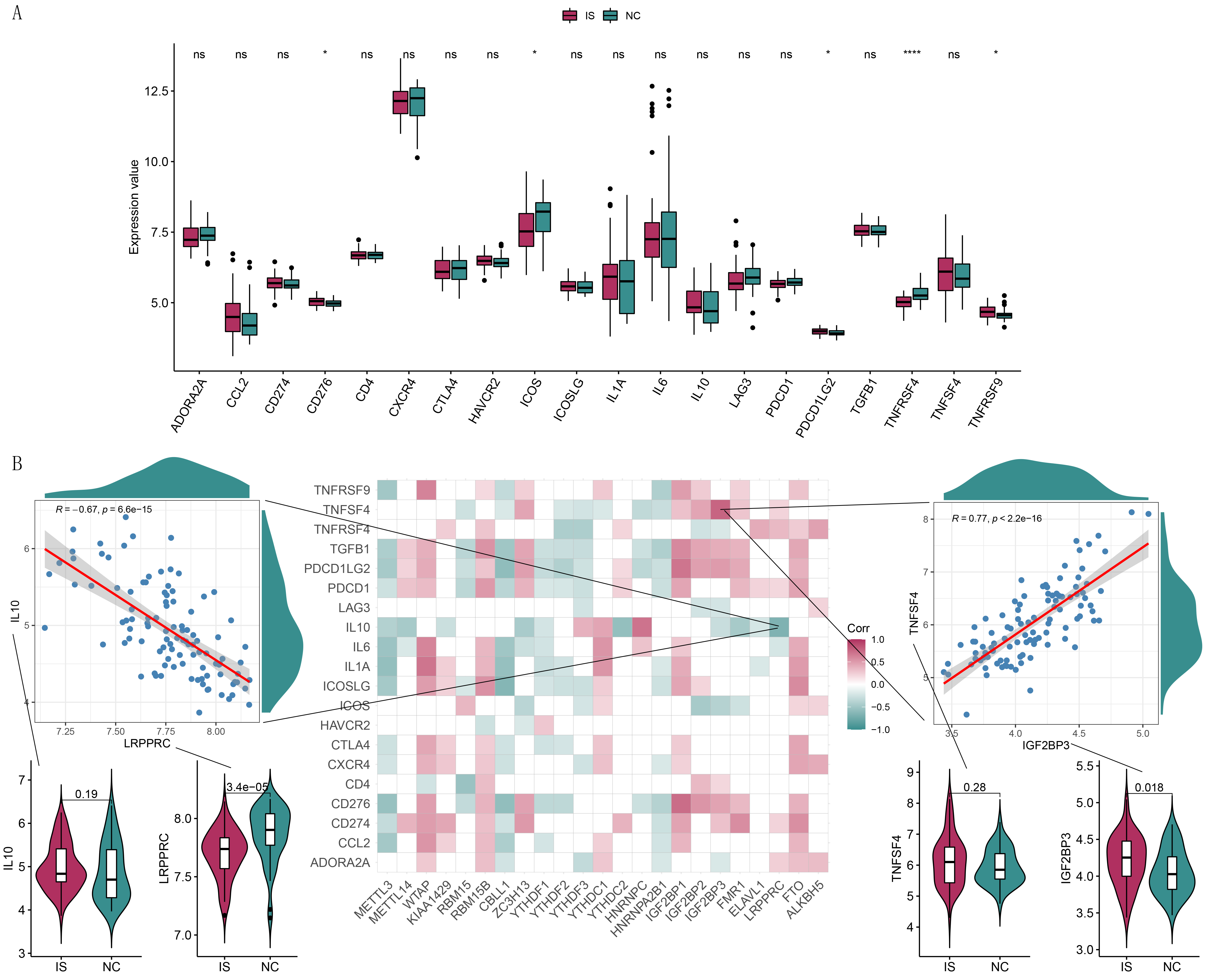

Supplement: Supplementary Figure 4 — The correlation between immune-related genes and m6A regulators. (A) The boxplot of the immune-related genes by the Wilcoxon test. There were significant differences in CD276, ICOS, PDCD1LG2, TNFRSF4, and TNFRSF9 between IS and NC groups. (B) Correlation plots show the correlation between the immune-related genes and each of the m6A regulators. The positive correlation between TNFSF4 and IGF2BP3 was higher at 0.77. The negative correlation between IL10 and LRPPRC was high at -0.67. IS, ischemic stroke. NC, normal. ns: value of p ≥ 0.05; *: value of p < 0.05; **: value of p < 0.01; ***: value of p < 0.001; ****: value of p < 0.0001. [file Image_4.TIF]

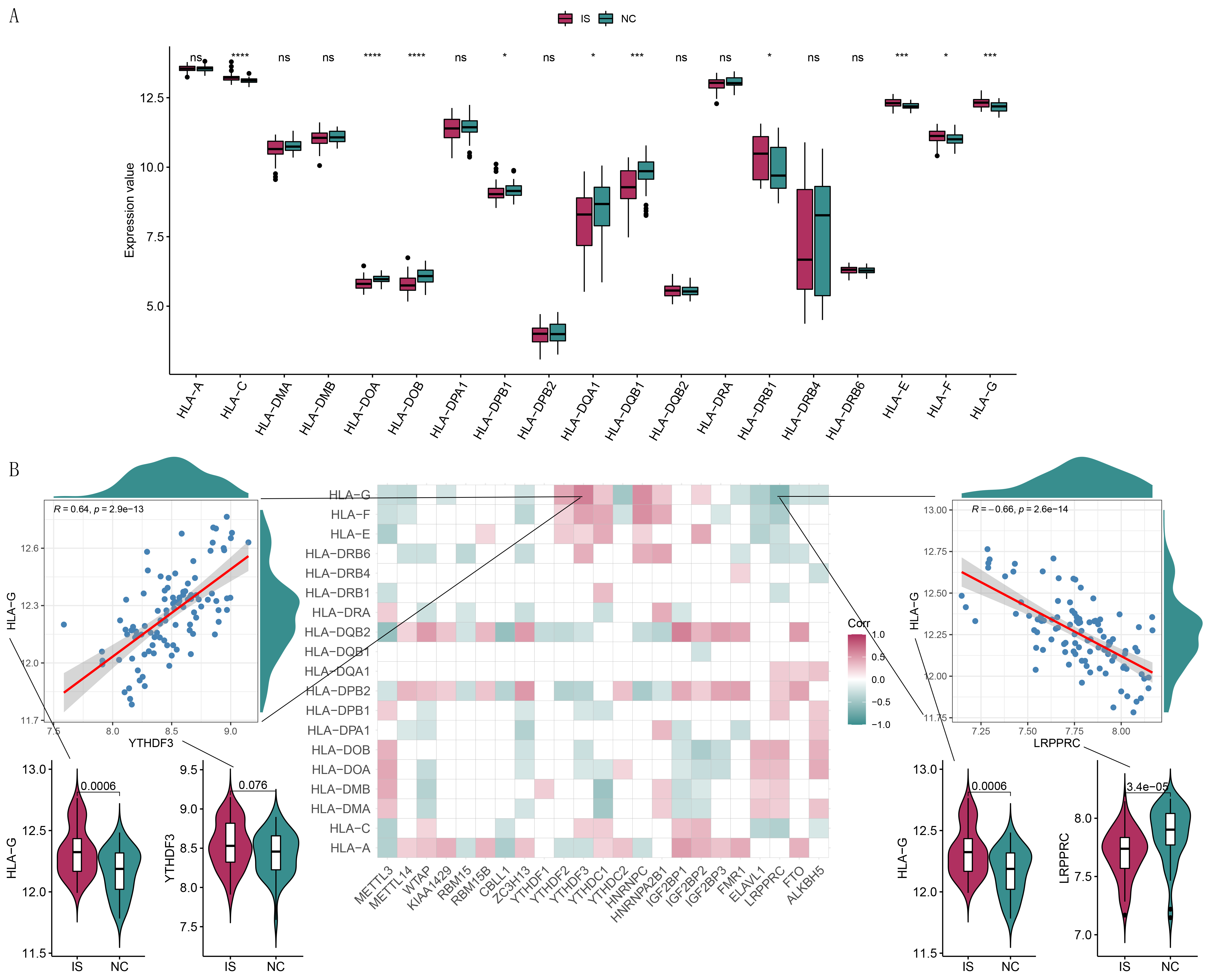

Supplement: Supplementary Figure 5 — The correlation between HLA-related genes and m6A regulators. (A) The boxplot of the HLA-related genes by the Wilcoxon test. There were significant differences in HLA−C, HLA−DOA, HLA−DOB, HLA−DPB1, HLA−DQA1, HLA−DQB1, HLA−DRB1, HLA−E, HLA−F, and HLA−G between IS and NC groups. (B) Correlation plots show the correlation between the HLA-related genes and each of the m6A regulators. The positive correlation between HLA-G and YTHDF3 was higher at 0.64, while the negative correlation between HLA-G and LRPPRC was higher at −0.66.HLA, human leukocyte antigen. IS, ischemic stroke. NC, normal. ns: p-value ≥ 0.05; *: value of p < 0.05; **: value of p < 0.01; ***: value of p < 0.001; and ****: value of p < 0.0001. [file Image_5.TIF]

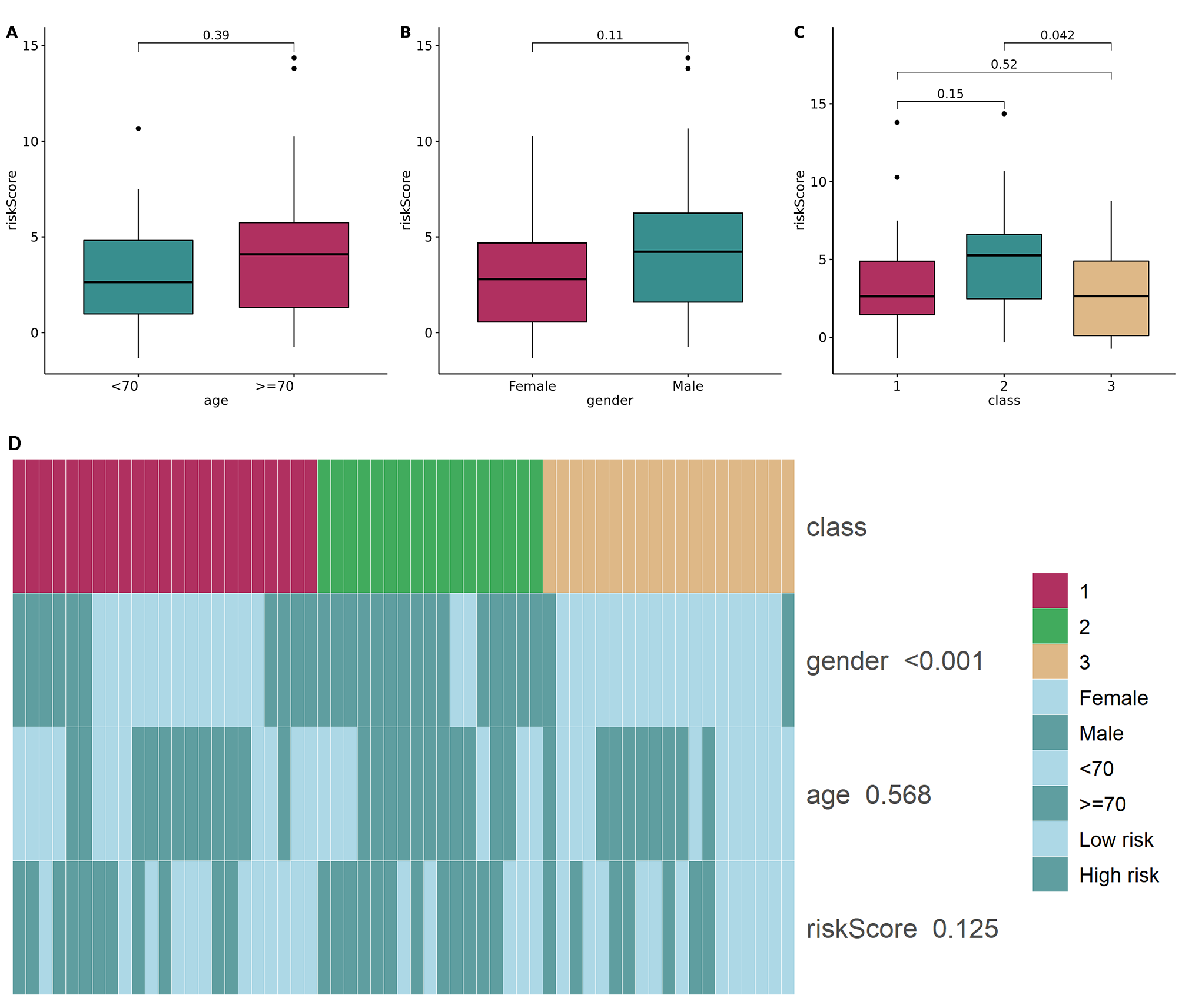

Supplement: Supplementary Figure 6 — The clinical characteristics of the situation between the different risk score (A–C) and m6A modification classes (D). [file Image_6.TIFF]

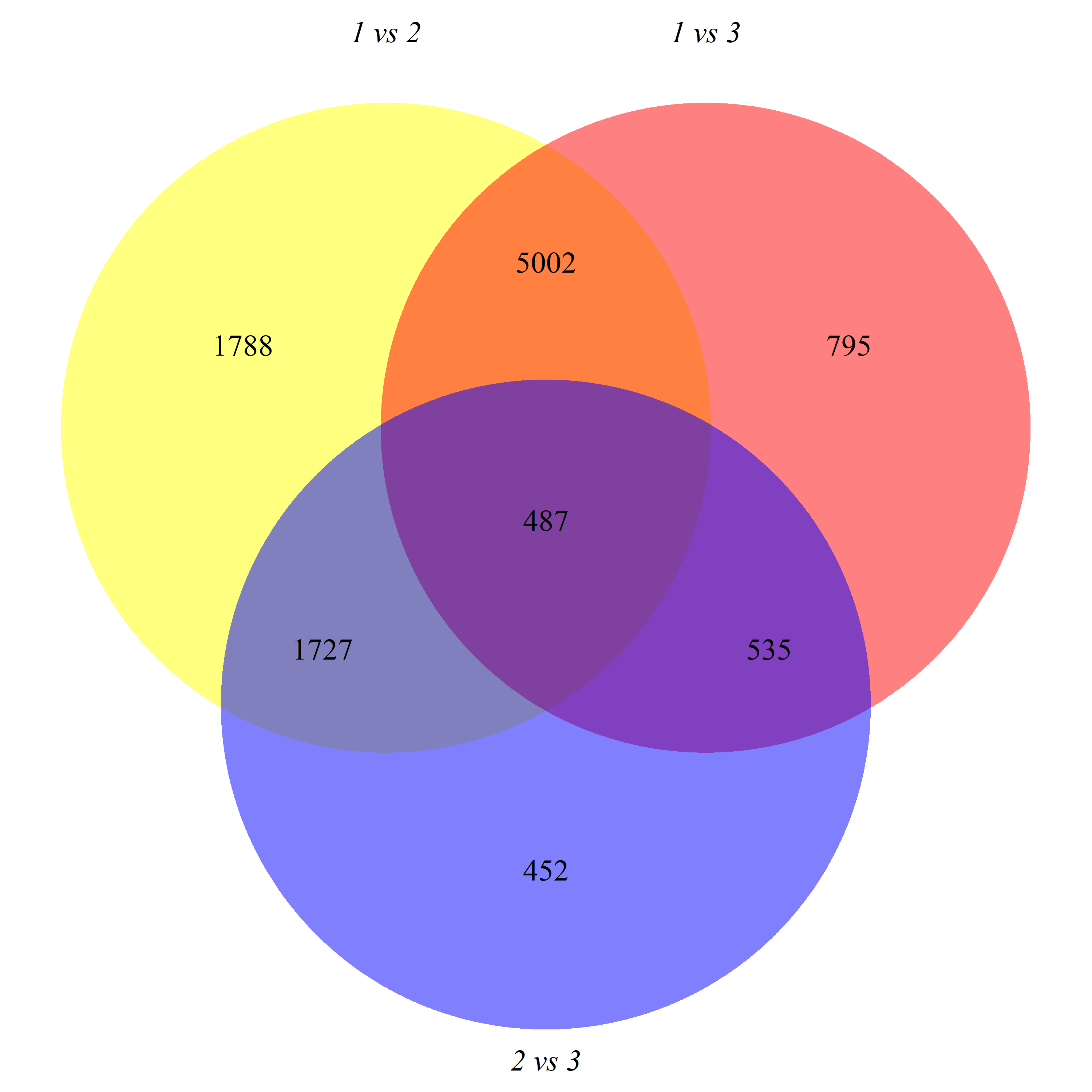

Supplement: Supplementary Figure 7 — Potential bifunctional features between the three m6A modification classes. [file Image_7.TIFF]
